# Wisdom of Crowds for Supporting the Safety Evaluation of Nanomaterials

**DOI:** 10.1021/acs.est.5c00841

**Published:** 2025-07-17

**Authors:** Laura Aliisa Saarimäki, Michele Fratello, Giusy del Giudice, Emanuele Di Lieto, Antreas Afantitis, Harri Alenius, Eliodoro Chiavazzo, Mary Gulumian, Piia Karisola, Iseult Lynch, Giulia Mancardi, Georgia Melagraki, Paolo Netti, Anastasios G. Papadiamantis, Willie Peijnenburg, Hélder A Santos, Tommaso Serchi, Mohammad-Ali Shahbazi, Tobias Stoeger, Eugenia Valsami-Jones, Paola Vivo, Ivana Vinković Vrček, Ulla Vogel, Peter Wick, David A. Winkler, Angela Serra, Dario Greco

**Affiliations:** † Finnish Hub for Development and Validation of Integrated Approaches (FHAIVE), Faculty of Medicine and Health Technology, 7840 Tampere University, Tampere 33520, Finland; ‡ Division of Pharmaceutical Biosciences, Faculty of Pharmacy, 3835 University of Helsinki, Helsinki 00790, Finland; § 443801 NovaMechanics Ltd., Nicosia 1065, Cyprus; ∥ Institute of Environmental Medicine, Karolinska Institutet, Stockholm 171 77, Sweden; ⊥ Human Microbiome (HUMI) Research Program, Medical Faculty, University of Helsinki, Helsinki 00290, Finland; # 19032 Politecnico di Torino, Corso Duca degli Abruzzi 24, Torino 10129, Italy; ∇ National Institute for Occupational Health, National Health Laboratory Services, Johannesburg 2001, South Africa; ○ School of Geography, Earth, and Environmental Sciences, 1724 University of Birmingham, Birmingham B15 2TT, U.K.; ◆ Division of Physical Sciences and Applications, Hellenic Military Academy, Vari 16673, Greece; ¶ Interdisciplinary Research Centre on Biomaterials-CRIB, University of Napoli Federico II, P.le Tecchio 80, Napoli 80125, Italy; †† Institute of Environmental Sciences, 4496 Leiden University, Leiden 2300 RA, The Netherlands; ‡‡ National Institute of Public Health and the Environment, Center for Safety of Products and Substances, Bilthoven 3720 BA, The Netherlands; §§ Department of Biomaterials and Biomedical Technology, The Personalized Medicine Research Institute (PRECISION), University Medical Center Groningen (UMCG), University of Groningen, Groningen 9700 RB, The Netherlands; ∥∥ Drug Research Program, Division of Pharmaceutical Chemistry and Technology, Faculty of Pharmacy, University of Helsinki, Helsinki 00790, Finland; ⊥⊥ Luxembourg Institute of Science and Technology, 5 Avenue des Hauts Fourneaux, Esch-sur-Alzette 4362, Luxembourg; ## Pneumology Center, Institute of Lung Health and Immunity, Helmholtz Center Munich, German and Research Center for Environmental Health, Neuherberg, German Center for Lung Research (DZL), Munich 85764, Germany; ∇∇ Solar Cells, Faculty of Engineering and Natural Sciences, Tampere University, P.O. Box 541, Tampere 33014, Finland; ○○ Institute for Medical Research and Occupational Health, Zagreb HR-10001, Croatia; ◆◆ 2686 National Research Centre for the Working Environment, Copenhagen O DK-2100, Denmark; ¶¶ Laboratory for Particles-Biology Interactions Swiss Federal Laboratories for Materials Science and Technology (Empa), Lerchenfeldstrasse 5, St. Gallen 9014, Switzerland; ††† La Trobe Institute of Molecular Science, La Trobe University, Bundoora 3086, Australia; ‡‡‡ Monash Institute of Pharmaceutical Sciences, Monash University, Parkville 3052, Australia; §§§ School of Pharmacy, University of Nottingham, Nottingham NG7 2QL, U.K.; ∥∥∥ Tampere Institute for Advanced Study, Tampere University, Tampere 33100, Finland

**Keywords:** wisdom of crowds, nanosafety, computational toxicology, engineered nanomaterials, new approach methodologies

## Abstract

The development of new approach methodologies (NAMs) to replace current *in vivo* testing for the safety assessment of engineered nanomaterials (ENMs) is hindered by the scarcity of validated experimental data for many ENMs. We introduce a framework to address this challenge by harnessing the collective expertise of professionals from multiple complementary and related fields (“wisdom of crowds” or WoC). By integrating expert insights, we aim to fill data gaps and generate consensus concern scores for diverse ENMs, thereby enhancing the predictive power of nanosafety computational models. Our investigation reveals an alignment between expert opinion and experimental data, providing robust estimations of concern levels. Building upon these findings, we employ predictive machine learning models trained on the newly defined concern scores, ENM descriptors, and gene expression profiles, to quantify potential harm across various toxicity end points. These models further reveal key genes potentially involved in underlying toxicity mechanisms. Notably, genes associated with metal ion homeostasis, inflammation, and oxidative stress emerge as predictors of ENM toxicity across diverse end points. This study showcases the value of integrating expert knowledge and computational modeling to support more efficient, mechanism-informed, and scalable safety assessment of nanomaterials in the rapidly evolving landscape of nanotechnology.

## Graphical abstract

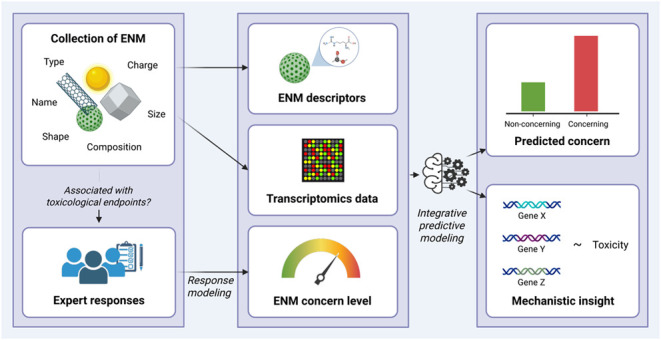

## Introduction

The rapid development of new nanomaterials continues to outpace our capacity to evaluate their safety, raising concerns about their safe use and health impacts, which may limit innovation in nanotechnology. This gap can be partly attributed to the resource-intensive nature of the current chemical safety assessment framework, as well as the complex nature of engineered nanomaterials (ENMs) and their interactions with biological systems. Tackling this complexity requires multidisciplinary expertise spanning fields such as chemistry, engineering, biology, exposure science, and toxicology. Moreover, the need to make chemical safety assessment faster and more effective has driven the development of nonanimal or new approach methodologies (NAMs), including diverse computational and predictive strategies.^1–3^ Among these, models based on mechanistic principles have emerged as valuable tools for comprehensive hazard characterization and to underpin the implementation of the Safe- and Sustainable-by-Design (SSbD) framework.^4,5^

Mechanistic toxicology models aim to enhance our understanding of chemical toxicity and to predict adverse effects by describing cascades of biological events that link exposure to adverse outcomes (AOs) such as cancer or skin sensitization, for example. However, the efficacy of these models is hindered by the lack of extensive biological end point data for the myriad forms of ENMs (chemical diversity and environmental transformations). Despite the growing number of data sets probing molecular mechanisms associated with human exposure to ENMs, public repositories still lack sufficient safety end point information to allow the generation of robust computational models to aid safety assessment of ENMs.

We hypothesized that complementary knowledge provided by experts could compensate for the lack of experimental nanosafety data for various classes of ENMs. Hence, we implemented a framework that harnesses the expertise of a panel of professionals from diverse nanosafety-related backgrounds to provide informed estimates of the relationships between the primary intrinsic characteristics of the materials in a recently curated collection^6^ and toxicological end points. The framework is based on the idea of the “wisdom of crowds” (WoC), whereby the collective expertise of a crowd is more informative and accurate than that of any individual. WoC and expert opinions have been applied across diverse fields for information-gathering and decision-making purposes, resulting in accurate predictions.^7–9^ In toxicology and medicine, previous examples employing expert opinions have been primarily focused on the Delphi method, in which consensus is reached through multiple iterations.^10–13^ This method has proven informative for applications such as policy definitions and characterization of optimal diagnostic criteria, treatment protocols and biomarker characteristics.^13–17^ We, on the other hand, applied a method that reaches consensus through a data-driven approach, enabling us to alleviate some of the challenges associated with the Delphi method, including those related to interpersonal influence, participant loss, and time the experts needed to dedicate to the task. We then integrated these expert-driven concern scores with computational modeling. By leveraging the collective insights of professionals, we aimed to generate reliable predictions of ENM toxicity and identify key molecular mechanisms underlying adverse effects.

This study presents the novel framework, evaluates its predictive power against experimental data, and demonstrates its potential for enhancing mechanistic toxicology models in the context of nanosafety.

## Materials and Methods

The methods used in this study are summarized below, providing an overview of the key experimental and analytical approaches. For a detailed description of the methodologies, including details about the data collection, model formulation, and data analyses, we refer to Supporting Materials and Methods in the Supporting Information.

### Expert Opinion Collection

A Wisdom of the Crowd (WoC) approach was applied to explore whether existing data gaps in nanotoxicology could be partially addressed by distilling expert knowledge into actionable outputs. Specifically, the goal was to assess the potential adverse effects of engineered nanomaterials (ENMs) by generating expert-derived concern levels for selected ENM-end point combinations as a proxy for missing empirical data. For this, 79 experts were invited, and 21 contributed responses, evaluating 134 ENMs across 18 toxicological end points (Supporting File 2 and Figure S1). Experts were asked to indicate potential connections between ENMs and end points. The heterogeneity of the participants was characterized by implementing a field of study analysis (see Field of study analysis, Supporting Information).

### Response Modeling and Concern Inference

Concern levels for each ENM and each pair of (ENM, end point) were estimated with a bayesian hierarchical model following a modeling approach similar to Whitehill et al.^18^ Furthermore, additional parameters, including end point difficulty and the level of expertise of each participant were also estimated. The model was optimized using Stochastic Variational Inference^19,20^ (SVI), implemented with the Pyro^21^ library in Python. Final concern labels were estimated by sampling 1000 times the fitted model, with uncertainty quantified based on the frequencies of the sampled values.

### Validation with Experimental Data

To evaluate result quality, we compared predicted concern labels with experimental data from 2896 ENM cell viability assays compiled from peer-reviewed studies.^22^ The data set includes annotations on ENMs (e.g., core material, coating, diameter, ζ-potential) and experimental conditions (e.g., cell type, concentration, exposure time, test type, positive controls). We focused on the most reported MTT assay.

Matching ENMs were identified based on core material, and the most frequent concern level was assigned to each type. We then compared the viability distribution of matched ENMs with their concern levels (see Figure S4, Supporting Information).

### Toxicogenomic and Descriptor Data Layers

ENM transcriptomic and physicochemical data were sourced from Saarimäki et al.^6,23^ and Gallud et al.^24^ available on GEO (GSE148705). Data aggregation focused on core material similarity, with human in vitro samples (294 samples, 7238 genes). Physicochemical descriptors included molecular/electronic structure attributes (del Giudice et al.^25,26^ ), omitting parameters available to experts (e.g., ζ-potential). Gene expression profiles were aggregated per ENM for machine learning analysis.

### Machine Learning Classifiers

We created multiple classification task instances based on data type (descriptors, gene expression, or both), gene expression aggregation, and prediction labels (ENM concern level for an end point or overall concern). Binary overall concern labels were assigned based on the most frequent value sampled by the survey model. Samples were also weighted based on the variability associated with the corresponding concern level (see Supporting Information for details).

For each task, we trained a gradient boosting classifier^27,28^ with 5000 trees, weighting samples by label uncertainty to prioritize the most certain cases. Model performance was evaluated using 5-fold cross-validation, repeated 10 times. This ensured robust evaluation with predictions derived from held-out data.

We applied two classification strategies: single-view and multiview integration. Single-view models used either physicochemical or gene expression data. For multiview integration, we tested both early and late integration approaches:^29^ early integration concatenated features before training, while late integration combined predictions from single-view models using a meta-classifier.

After training, we identified the top 10 most relevant features (genes) based on information gain across classification tasks. The most frequently selected features are reported (Supporting File 1).

### Interactive Data Viewer

A Shiny-based interactive viewer was developed for data exploration. The tool, along with all related code, is available on GitHub at https://github.com/fhaive/wisdom_of_the_crowds.

## Results and Discussion

### The Expert Survey Reveals Distinct Levels of Knowledge of the ENMs

The survey results were examined to evaluate the overall perception of the hazards related to each ENM. The responses of the experts varied in coverage across the ENMs and the end points, with some experts limiting their responses only to selected ENM-end point pairs while others provided estimates across the whole collection (see Figure S2A, Supporting Information). In other words, not all experts felt confident to give informed opinions on the relationships between all ENMs and all potential AOs.

The number of definitive (yes/no) answers per ENMs/end point pair ranged from 3 to 16, revealing clusters of low-coverage ENMs and end points (see Figure S2B, Supporting Information). More specifically, nanodiamonds with different functionalizations (ND_X), graphite nanofibers (GNF), aminated graphite oxide (GONH_2_), and tungsten carbide-based (WC) ENMs fall into a distinct cluster of low coverage across all end points. The total number of responses over the end points was highest for cytotoxicity (1504) and lowest for neuro-, nephro-, and cardiovascular toxicity (707, 712, and 736, respectively).

These numbers are affected by factors such as the background and expertise of the experts (see Supporting File 1 for expert characterization), popularity of the ENMs in research, relevance of the end points for the ENMs under real-life exposure scenarios, and many others. Although the experts were asked to provide the responses based on their experience and expertise without extensive literature searches, the results ought to be affected by the data and literature available. The literature, on the other hand, is likely biased with larger data sets for ENMs with high production volume and hazard potential, and the assessment of end points of highest regulatory relevance or based on the ease of assessment (e.g., *in vitro* cytotoxicity),^30^ resulting in the experts to be generally more knowledgeable in these aspects. This is clear from the high number of responses for cytotoxicity, an assay often used as the foundation for further experimentation, and certain ENM-end point pairs, such as different types of asbestos fibers and lung fibrosis (see Figure S2B, Supporting Information). While asbestos is not an ENM, it was included in our analysis due to its widespread use as a positive control in experiments and its many parallels with ENM toxicity.^31–33^

Although it is reasonable to focus resources on the chemicals with the highest potential risk either through production volume, hazard, or the most likely routes of exposure, the lack of data for the less characterized ENMs and exposures does not support their safe use. Similarly, the publication bias toward positive relationships is well established, and likely affects the field of nanosafety as well.^34^ This, in turn, makes the identification of true negatives challenging, and requires consideration of thresholds of effect since everything is toxic at sufficient concentration.

### Expert Agreement and End Point Difficulty

While the raw responses elucidate the different levels of knowledge the experts have on specific ENM-end point pairs and suggest potential hazard trends, the comparison and interpretation of the results is demanding due to imbalances in the responses. To address this, we hypothesized that a consensus concern score could be derived from the expert input using a probabilistic model. This model estimates both the intrinsic concern level of each ENM and its contextualized concern for specific end points.

The model uses two sets of parameters to represent the level of concern: a measurement of how consistently the responses of the expert align with the consensus (agreement score); and a difficulty score characterizing each end point in terms of the likelihood of finding consensus, making end points dominated by opposing responses more “difficult”, hence reflecting different opinions of the experts.

We characterized the two scores (agreement and difficulty) for each expert and end point, respectively (Figure S3A,C). In the case of the expert agreement scores, higher values indicate a greater likelihood of accurate labeling in alignment with the consensus. Notably, scores can take negative values, implying systematic errors in labeling possibly due to implicit biases or malicious behavior. However, our analysis showed that all modes were >0, suggesting a lack of malicious intent despite significant variability in expert agreement scores. Similarly, the values in the difficulty score reflect the ease of reaching consensus, with end points scoring higher having greater levels of expert agreement. Further analysis showed no clear correlation between the quantity of responses and the agreement scores or difficulty scores (see Figure S3B,D, Supporting Information). Experts providing more answers did not consistently align with the consensus, and *vice versa*, and the number of answers received for each end point did not seem to reflect its inferred difficulty.

End points such as neurotoxicity, skin sensitization, and cytotoxicity had higher agreement, likely due to clearer manifestations, established *in vitro* assays, and well-defined biomarkers.^35–39^ In contrast, lung fibrosis was the most challenging, given its complex pathophysiology, need for long-term exposure studies, and absence of standardized biomarkers. Despite lung-related outcomes being one of the most studied in nanosafety,^30^ expert consensus remained low, underscoring the difficulty of assessing chronic effects in acute experimental setups.

These findings suggest an emerging consensus on a bottom-up approach. Moreover, they emphasize the need for interdisciplinary collaboration in nanosafety research, as ENM interactions with biological systems remain highly complex and sometimes contradictory.

### Overall Concern Level Analysis Reveals Three Concern Groups

The inferred parameters of the probabilistic model define an overall concern score summarizing expert-perceived concern levels for each ENM. Based on this score, ENMs cluster broadly into three concern categories (Figure 1A), which also align with their core compositions (Figure 1B).

**Figure 1:**
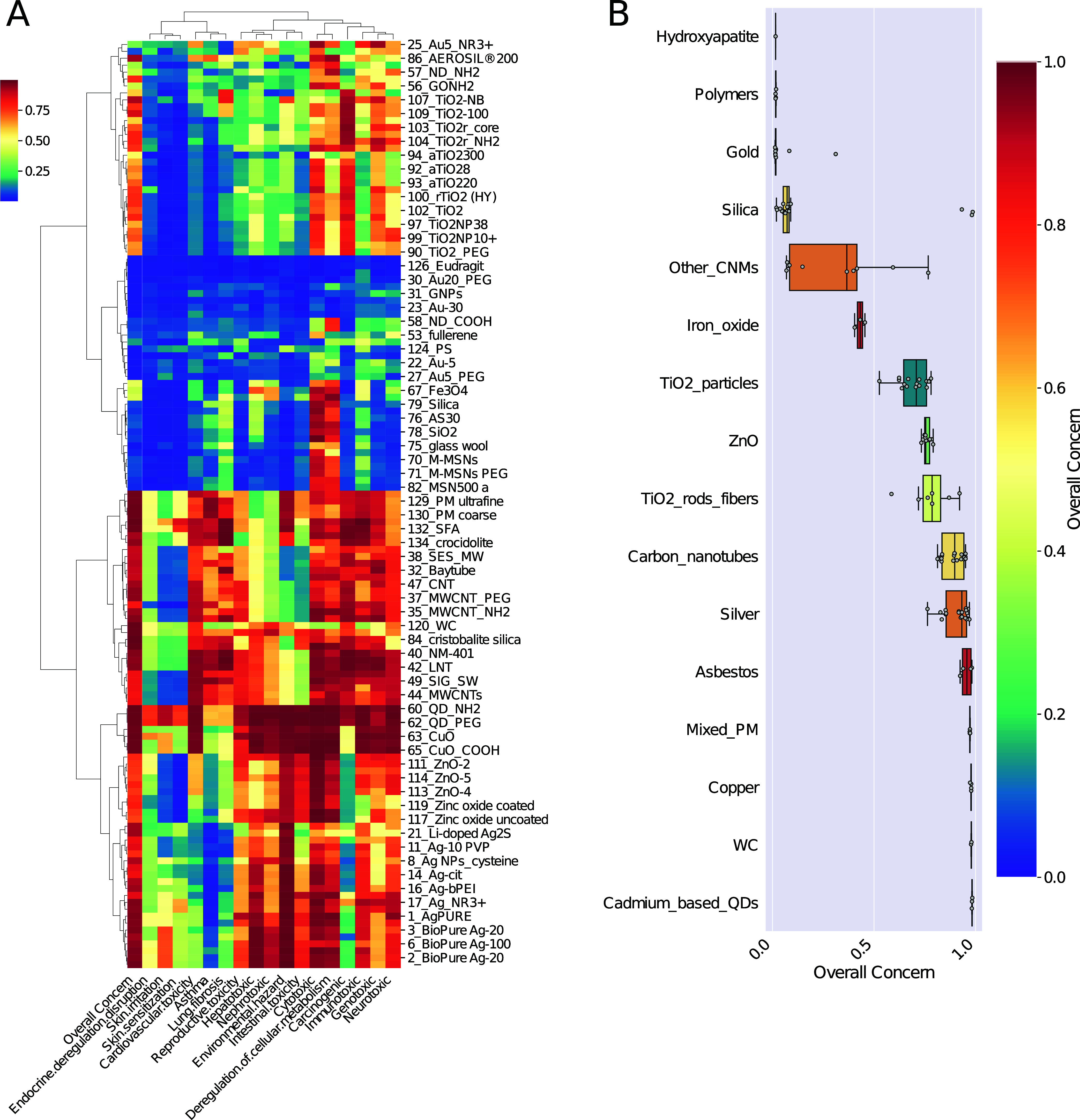
(A) Probabilistic concern scores for the studied ENMs and end points. Red indicates high levels of concern, while blue indicates low levels of concern. The ENMs are grouped based on the concern scores across the end points. The overall concern shows three distinct clusters by color (leftmost column). (B) Overall concern shown by the ENM category. ENMs were grouped by the core material and type.

ENMs in the low-concern category include polymers, hydroxyapatite, gold, and silica, though some outliers into the other categories suggest that factors like crystallinity, particle size, and surface functionalization may influence their hazard potential. Hydroxyapatite nanoparticles (NPs) are commonly used for drug delivery and bone regeneration purposes due to their high biocompatibility.^40–42^ Likewise, Eudragit particles (also under polymers) are used for drug delivery, while gold NPs have found various biomedical applications due to their inert nature.^43,44^ Although most of the gold-based NPs fell into the low concern category, ammonium-functionalized gold NPs (AuX_NR3) exhibited intermediate concern, aligning with previous studies linking said surface modifications to increased cytotoxicity.^24^

The intermediate-concern group comprises iron oxide NPs, titanium dioxide (TiO_2_) particles and rods, and various carbon nanomaterials as defined under the label “other carbon nanomaterials”. The higher variability among these ENMs may imply that there is no inherent association between the core material and its concern level, but it is more broadly affected by the physicochemical characteristics, including surface functionalization. The intermediate concern is reflected in the literature on the toxicity of TiO_2_ NPs and -rods. TiO_2_ particles have been long considered poorly soluble and of low toxicity, resulting in various applications ranging from cosmetics and food additives to paints and dyes.^45^ While the evidence pointing toward the safety of TiO_2_ was largely derived for particles in the fine range (100 nm to 2.5 μm), the idea has been more recently challenged by increasing reports of the harmful effects of nanosized TiO_2_ in various organisms.^46–48^ Several studies have indicated the potential of these particles to induce oxidative stress, inflammation, and cellular damage due to their ability to generate reactive oxygen species, while others have reported contradictory findings.^49–51^ Concerns over genotoxicity also prompted the recent EU ban on the use of TiO_2_ as a food additive (E171, in which up to 50% of the particles are nanosized).^52^ However, the overall evidence on the toxicity of TiO_2_ is varied and often skewed by extremely high exposure doses.^47^ Despite the broad applications of these particles, epidemiological data on the effects of TiO_2_ exposure is largely missing, due in large part to their topical application via suncreams (where animal testing has been forbidden in the EU since 1998) and assumptions regarding the effective barrier properties of the skin resulting in low exposure.

ENMs in the high-concern group (zinc oxide, tungsten carbide, silver, copper, carbon nanotubes, asbestos, mixed particulate matter, and cadmium-based quantum dots (QDs)) are generally classified as reasonably soluble. The highest concerns were associated with cadmium-based QDs and copper oxide (CuO) NPs, both linked to cytotoxicity through the release of free metal ions.^24,53,54^ Interestingly, as opposed to the low-concern category, there is little variation among the scores of this group, possibly implying that these materials are considered inherently harmful regardless of their functionalization and primary characteristics. While moderate variation is observed between end points, the high overall concern is driven by multiple end points with high concern levels (Figure 1A).

### Concern Levels Are Consistent with Experimental Data

We then evaluated whether our predicted concern scores agreed with experimental data. Due to cell viability being the only end point with a large enough curated collection available,^22^ we focused on a comparison between cytotoxicity concern scores and cell viability data from Labouta et al..

In general, a matching trend between our computed concern scores and experimental data was observed, with higher concern scores being associated with lower viability, or at least with highly variable measurements (see Figure S4, Supporting Information).

While these results show consistency between the computed concern scores and the experimental evidence, the challenges in matching ENMs between multiple data sets complicates their interpretation. Moreover, the cell viability data is derived from diverse experimental conditions with multiple dose ranges, exposure times, and biological systems.^22^ The exposure details and conditions, such as ENM characteristics, coating/functionalization, cell type, dose, and exposure time, influence cell viability and likely explain the high variability in the data. Likewise, assay inference by ENMs is an often-overlooked issue that may introduce biases into this type of data.^55^

Although these results are suggestive/correlative rather than an absolute validation, this comparison allowed a systematic assessment of our methodology. We confirmed that the expectations of those with extensive knowledge and experience in the subject matter are consistent with experimental results and thus can be considered a source of knowledge when experimental results are not available, or abundant enough. Optimally, the predicted concern scores would be benchmarked against comprehensive experimental data sets. However, such data are scarce for most of ENMs and, where available, are often limited to specific conditions, restricting their application for computational approaches and systematic evaluation. Broader, well-curated data sets covering diverse toxicity end points would enable more robust assessment of expert-derived concern scores and strengthen confidence in the WoC approach. Likewise, this highlights the need for caution in terms of nomenclature and data reporting in nanobio interaction literature.^56^

Overall, our results show that the complementary expertise of the multidisciplinary panel of experts can converge into robust consensus that aligns with experimental evidence. Hence, we further hypothesized that the consensus expert opinions could be used to support the classification and prioritization of ENMs, and to derive relevant proxies of ENM concern, paving the way for predictive NAMs. Nevertheless, predictions for these end points should be interpreted cautiously, and further experimental validation is essential to extend the applicability and robustness of the developed models across a broader range of nanosafety concerns.

### Individual End Points Show Different Confidence Levels

To assist the interpretation of the concern scores, we inferred a binary concern label (concerning or nonconcerning) for each ENM with respect to each end point. Due to the probabilistic nature of the model, the inferences are associated with a degree of variability/uncertainty. We quantified the variability by the relative frequencies of each label outcome in 1000 repeated samples from the posterior distribution of the concern labels estimated by the probabilistic model. The distribution of the concern labels and their variability across the end points is depicted in Figure S5.

The distribution of “concerning” and “non-concerning” ENMs varies between the end points. For instance, cytotoxicity and deregulation of cellular metabolism are unbalanced toward the concerning ENMs, suggesting that most of the ENMs in the collection are potentially associated with these end points. On the other hand, skin irritation and skin sensitization are more unbalanced toward nonconcerning ENMs, while the rest of the end points are more balanced across the two labels. Moreover, the number of highly uncertain ENMs is not evenly distributed across the end points. For example, asthma, carcinogenicity, cardiovascular toxicity and environmental hazard have a smaller number of ENMs associated with high uncertainty (<0.6 certainty in the labeling), while end points such as hepatotoxicity, neurotoxicity and lung fibrosis have higher numbers of uncertain ENMs. This lack of consensus can be traced back to the survey data. All the ENM-end point pairs with a highly uncertain label correspond to pairs in which the expert annotations are evenly split between the yes/no answers, making the data agree perfectly with the prior distribution of labels. This results in a lack of evidence to deviate from the prior label distribution. These instances could reflect cases where experimental data is highly controversial or largely absent, and the expert judgment is solely based on impressions of the material type and characteristics. Such cases illustrate the utility of the WoC framework both by supporting informed predictions where existing knowledge is sufficient as well as by highlighting areas where the current evidence base is too weak or fragmented to support consensus. Through the identification of such knowledge gaps, the approach informs prioritization of future experimental efforts, indicating where additional data collection is necessary to reach a critical mass of evidence.

### Physicochemical Properties Are Correlated with the WoC Predictions

Following the definition of the concern level categories for each end point using the data from the WoC survey, we trained machine learning classifiers to predict the hazard potential of ENMs. We employed several strategies, including single view (physicochemical characteristics or gene expression alone) or multiview (both features combined) with multiple data aggregation strategies. This allowed us to evaluate specific features linked to ENM hazard potential while gaining mechanistic insights through gene expression data. These classifiers could support hazard assessment for novel or untested ENMs by predicting toxic potential from available descriptors. Indeed, reliable predictions can be expected for materials whose descriptor profiles are well represented in the current training set. However, given the highly heterogeneous nature of ENMs, it remains difficult to characterize a fixed applicability domain. Importantly, the physicochemical descriptors used here (e.g., molecular and electronic structure properties) can be calculated for other ENMs, enabling model extensibility beyond this data set. The insight gained could also inform grouping and prioritization by identifying toxicity-relevant features and biomarkers as proxies of toxicity.

The classifier trained on physicochemical descriptors alone reached the highest ROC-AUC for all end points, followed by the integrated model using a late integration strategy (see Figure S6, Supporting Information). The performance of the classifiers was also dependent on the end point, with the prediction of intestinal toxicity reaching top ROC-AUC, while skin sensitization and endocrine disruption were the most challenging outcomes to predict (see Figure S6, Supporting Information).

The combination of physicochemical properties and exposure characteristics drive the toxicity of ENMs. However, previous studies have indicated that the primary physicochemical characteristics, such as core material and size alone are not robust predictors of toxicity.^57^ Here, ENM descriptors were used instead of the primary characteristics. Although many of these descriptors are heavily influenced by the primary features that were also reported to the experts, complete blinding would be impossible, as the ENM type and name itself often provides some information on the properties of each ENM.

Despite the best performance arising from the physicochemical descriptors, the models generated on these characteristics alone provide a mere black box view of the toxic potential without any insights into the mode of action. It may inform on the properties that need to be altered to develop safer ENMs but gives no data on the molecular interactions between ENMs and biological systems, leaving the mechanisms of toxicity uncovered. Understanding the relationship between physicochemical properties and toxicity mechanisms can allow the rational development of safer ENMs while also generating valuable information for the development of new testing strategies for nanosafety assessment.^5,58^

Hence, we hypothesized that the transcriptional changes induced by the exposures could serve as proxies of ENM toxicity regardless of the exposure system. These gene proxies could then elucidate on the underlying molecular mechanisms behind ENM toxicity, supporting the identification of molecular initiating events for ENM-induced adverse outcome pathways and the development of gene based targeted assays while also expanding the general understanding of ENM toxicity.^57,59,60^

### Integration of Transcriptomic Characteristics Highlights Mechanisms of ENM Toxicity

Models of chemical-biological interactions inform the mechanisms of toxicity and can thus support the development of SSbD chemicals and materials.^5^ Given the multiple experimental conditions, we sought an underlying molecular mechanism that could describe the toxicity potential of ENMs regardless of the experimental setup. We identified the gene-based features with the highest influence on the classification task. The features were ranked by their relevance, i.e., their ability to discriminate between the concern categories for each end point.

Focusing on the 10 most important genes for each end point, we identified a total of 103 genes (see Table S1, Supporting Information), most of which were specific to individual end points (75 genes), while the remaining 28 genes were shared by two or more (Figure 2). Among these, we observed several members of the metallothionein (MT) family which encode for proteins that bind both physiological and xenobiotic metals, maintaining metal homeostasis and participating in cellular detoxification and protection against oxidative stress.^61–63^ MT1F, and MT2A ranked among the top genes predicting environmental hazard, intestinal toxicity, nephrotoxicity, and skin irritation while MT1G was also predictive of hepatotoxicity and reproductive toxicity. The expression of MTs has been previously correlated with SLC30A1, a gene coding for zinc transporter 1,^64^ which ranked among the top features for environmental hazard, hepatotoxicity, intestinal toxicity and nephrotoxicity (Figure 2).

**Figure 2:**
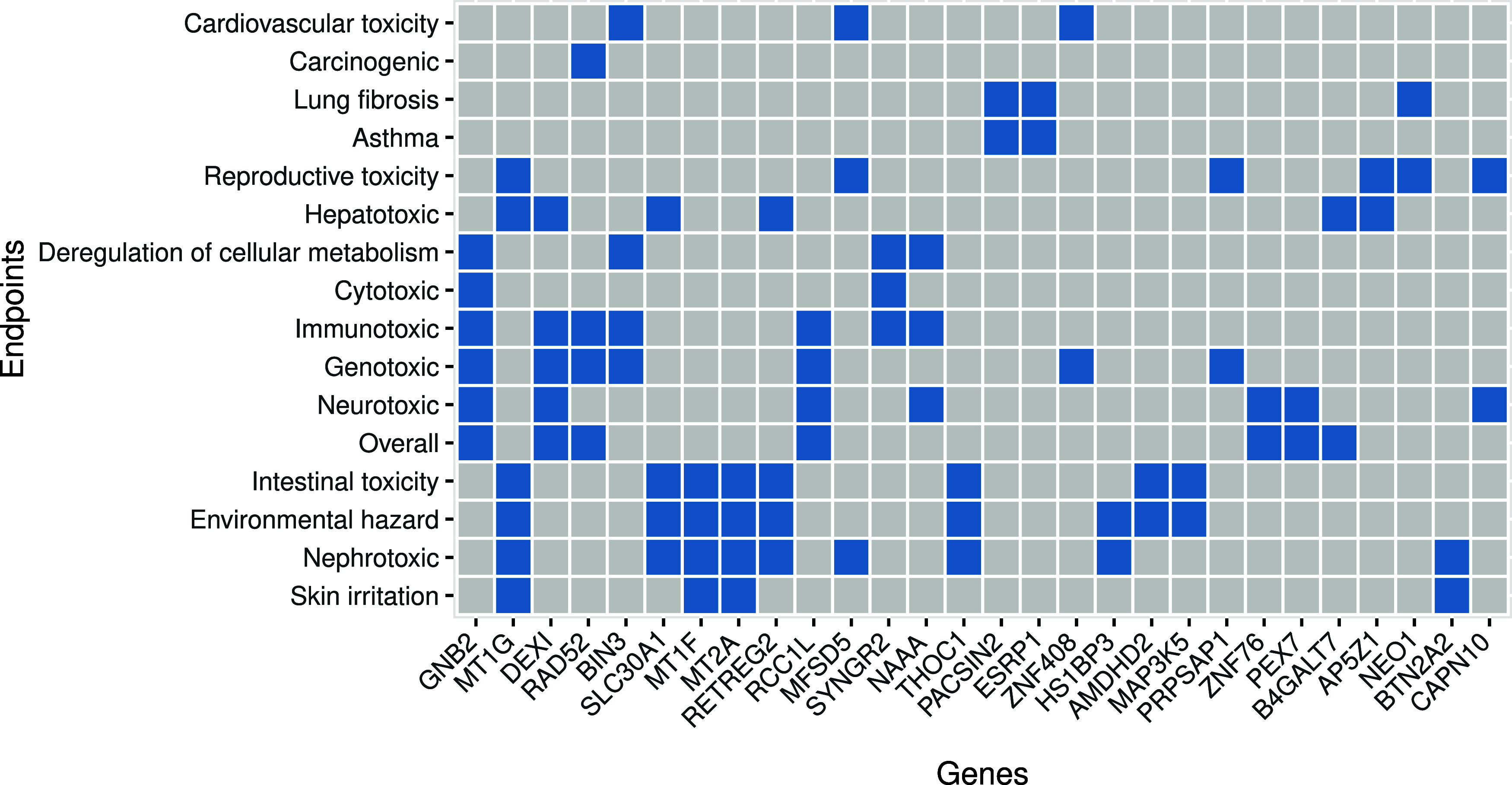
Relevant genes shared by two or more end points (classifier based on late integration strategy). Blue indicates the gene ranks among the top 10 gene features for the end point.

The cooperation of MTs and SLC30A1 for multiple end points suggests mechanisms related to the regulation of cellular zinc homeostasis.^65^ SLC30A1 is transcriptionally upregulated by high zinc levels.^64^ While this response has been associated with both physiological and xenobiotic zinc, the emergence of these genes in the context of this specific data set suggests toxicity mechanisms involving physiological zinc, given the small number of zinc-based ENMs in the collection. Indeed, zinc serves as an important regulator of cellular signaling and gene expression, interacting with various transcription factors (TFs).^66,67^

The important role of zinc was further highlighted by several genes encoding for members of the C2H2 zinc finger family (ZNF408, ZNF76, ZNF189, ZNF157) (Table S1), which we have recently reported as important regulators of toxicologically relevant genes activated by ENM exposure, with high levels of conservation across species.^25^ Although the data used in this study is limited to human cells, these findings linking to zinc homeostasis could also extend to other species given the conservation of these TFs and the members of the MT family.^25,68^ The overrepresentation of MTs and other players involved in metal homeostasis may also point toward toxicity mechanisms based on ion release. While this has been suggested as a major driver of metal ENM toxicity, our previous analysis of the same data collection did not fully support this idea.^25^ Although this cannot be determined definitively without further experimentation, the signature observed here could also suggest mechanisms related to oxidative stress induction and disruption of zinc homeostasis through other means.

In addition to zinc homeostasis and oxidative stress, other potential mechanisms arise. For instance, ESRP1 was found to be predictive of both asthma and lung fibrosis. ESRP1 has been suggested as the driver of epithelial to mesenchymal transition (EMT) in certain cancers.^69,70^ EMT, however, is also an important factor in fibrosis and its role in asthmatic airway remodeling has been clarified.^71–73^ ARNT2 emerged as a relevant feature for asthma (see Table S1, Supporting Information). This gene codes for a protein that acts as a TF which associates with other proteins to regulate gene expression.^74,75^ Moreover, ARNT2 belongs to the hypoxia inducible factor (HIF) family, whose best-characterized member HIF-1α has been linked to asthma and allergic airway inflammation in various studies.^76–79^ Similarly, CD14 stood out among the relevant genes for lung fibrosis. CD14 is mostly expressed by monocytes, and it primarily mediates innate immune responses.^80^ Increased CD14 expression in the lungs generally indicates recruitment of monocytes into the lungs, a clarified driver of lung fibrosis.^81,82^ Moreover, previous studies have shown higher CD14 expression in myeloid cells extracted from the lungs of patients suffering from idiopathic pulmonary fibrosis than in healthy controls.^83^

RAD52, coding for a DNA repair protein,^84^ appeared among the top features for genotoxicity, carcinogenicity, immunotoxicity and overall hazard (Figure 2). All these end points are associated with DNA damage and DNA repair either directly or indirectly. While the results suggest that potential DNA damage response might be captured in the transcriptomics data set, the mechanisms remain unclear. RAD52 has been associated with oxidative stress,^85^ yet the other genes postulated to be associated with oxidative stress are not linked to the expression of RAD52 in the data. Furthermore, ENMs are known to induce membrane damage^86^ and they have been suggested to disturb the cytoskeleton, which can further interfere with basic cellular functions, including transportation and cell division.^87,88^ Damage to organelles, such as lysosomes, endoplasmic reticulum, and mitochondria disturb cellular metabolism and induce oxidative stress. This, in turn, impairs cellular functions and can result in DNA damage, cell cycle arrest, apoptosis and inflammation.

Interestingly, none of the genes discussed here in relation to oxidative stress arise among the top features for neurotoxicity despite a clear mechanistic link.^89^ Instead, PEX7 encoding a protein involved in the function of peroxisomes was among the important features for neurotoxicity (Figure 2). Peroxisomes are specialized organelles carrying out oxidative functions while also scavenging reactive oxygen species.^90^ Of note, peroxisomal dysfunction has been linked to neurodegenerative disorders and cellular aging.^91^

While the discussion here revolves primarily around individual genes, they highlight some important mechanisms behind various ENM-related toxicity end points, suggesting that indications of these processes could be captured regardless of the test system and experimental setup. Hence, these genes could serve as proxies of ENM hazard, while exposure associated risks are to be defined on a case-to-case basis. The identification of these types of gene markers paves the way for the development of NAMs for the screening of potential hazards preemptively, while also informing on the mechanism of ENM-biomolecule interactions. This further supports the prioritization of ENMs and end points for more thorough safety assessment.

## Supplementary Material





## Notes

Details about the collection of expert opinions; description of the analysis of the field of studies; formulation of the response modeling and inference of the concern levels; details of the comparison of concern levels with experimental data; description of the toxicogenomic and nanomaterial descriptors; details of training and validation of machine learning classifiers; implementation details of the interactive data viewer; number of distinct nanomaterials under each category defined by core material/type; comparison of predicted cytotoxicity concern levels and experimental evidence derived from cell viability assays; top 10 most relevant genes for each endpoint (PDF)
Instructions, and ENM endpoints (XLSX)
