# Supplementary material for: Wisdom of Crowds for Supporting the Safety Evaluation of Nanomaterials
Source: Environ Sci Technol. 2025 Jul 17;59(29):14969–80. doi: 10.1021/acs.est.5c00841 (PMC12312164; doi:10.1021/acs.est.5c00841)
Supplement: Supplementary file 1 [file es5c00841_si_001.pdf]

Supporting Information

Wisdom of Crowds for Supporting the Safety Evaluation of Nanomaterials

Laura Aliisa Saarimäki[1,2,†], Michele Fratello[1,†], Giusy del Giudice[1,2], Emanuele Di Lieto[1], Antreas Afantitis[3], Harri Alenius[4,5], Eliodoro Chiavazzo[6], Mary Gulumian[7], Piia Karisola[5], Iseult Lynch[8], Giulia Mancardi[6], Georgia Melagraki[9], Paolo Netti[10], Anastasios G. Papadiamantis[3,8], Willie Peijnenburg[11,12], Hélder A. Santos[13,14], Tommaso Serchi[15], Mohammad-Ali Shahbazi[13], Tobias Stoeger[16], Eugenia Valsami-Jones[8], Paola Vivo[17], Ivana Vinković Vrček[18], Ulla Vogel[19], Peter Wick[20], David A. Winkler[21,22,23], Angela Serra[1,2,24], Dario Greco[1,2,]*

[1] *Finnish Hub for Development and Validation of Integrated Approaches (FHAIVE), Faculty of Medicine and Health Technology, Tampere University, Tampere, 33520, Finland*

[2] *Division of Pharmaceutical Biosciences, Faculty of Pharmacy, University of Helsinki, Helsinki, 00790, Finland*

[3] *NovaMechanics Ltd, Nicosia, 1065, Cyprus*

[4] *Institute of Environmental Medicine, Karolinska Institutet, Stockholm, 171 77, Sweden*

[5] *Human Microbiome (HUMI) Research Program, Medical Faculty, University of Helsinki, Helsinki, 00290, Finland*

[6] *Politecnico di Torino, Corso Duca degli Abruzzi 24, Torino, 10129, Italy*

[7] *National Institute for Occupational Health, National Health Laboratory Services, Johannesburg, 2001, South Africa*

[8] *School of Geography, Earth, and Environmental Sciences, University of Birmingham, Birmingham, B15 2TT, UK*

[9] *Division of Physical Sciences and Applications, Hellenic Military Academy, Vari, 16673, Greece*

[10] *Interdisciplinary Research Centre on Biomaterials-CRIB, University of Napoli Federico II, P.le Tecchio 80, Napoli, 80125, Italy*

[11] *Institute of Environmental Sciences, Leiden University, Leiden, 2300 RA, The Netherlands*

[12] *National Institute of Public Health and the Environment, Center for Safety of Products and Substances, Bilthoven, 3720 BA, The Netherlands*

[13] Department of Biomaterials and Biomedical Technology, The Personalized Medicine Research Institute (PRECISION), University Medical Center Groningen (UMCG), University of Groningen, Groningen, 9700 RB, The Netherlands

[14] Drug Research Program, Division of Pharmaceutical Chemistry and Technology, Faculty of Pharmacy, University of Helsinki, Helsinki, 00790, Finland

[15] Luxembourg Institute of Science and Technology, 5 Avenue des Hauts Fourneaux, Esch-sur-Alzette, 4362, Luxembourg

[16] Pneumology Center, Institute of Lung Health and Immunity, Helmholtz Center Munich, German and Research Center for Environmental Health, Neuherberg, German Center for Lung Research (DZL), Munich, 85764, Germany

[17] Solar Cells, Faculty of Engineering and Natural Sciences, Tampere University, P.O. Box 541, Tampere, 33014, Finland

[18] Institute for Medical Research and Occupational Health, Zagreb, HR-10001, Croatia

[19] National Research Centre for the Working Environment, Copenhagen O, DK-2100, Denmark

[20] Laboratory for Particles-Biology Interactions Swiss Federal Laboratories for Materials Science and Technology (Empa), Lerchenfeldstrasse 5, St. Gallen, 9014, Switzerland

[21] La Trobe Institute of Molecular Science, la Trobe University, Bundoora, 3086, Australia.

[22] Monash Institute of Pharmaceutical Sciences, Monash University, Parkville, 3052, Australia.

[23] School of Pharmacy, University of Nottingham, Nottingham, NG7 2QL, UK

[24] Tampere Institute for Advanced Study, Tampere University, Tampere, 33100, Finland

† *Equal contributions*

** Corresponding author: dario.greco@tuni.fi*

Number of pages: 19

Number of figures: 7

Number of tables: 2 (1 table in excel)

Contents

Supporting Information 1
Supporting materials and methods 3
Collection of expert opinions 3
Field of study analysis 4
Response modeling and inference of concern levels 4
Comparison of the concern levels with experimental data 6
Toxicogenomic and descriptor data layers 6
Machine learning classifiers 7
Interactive viewer implementation 9
Supporting results 9
The expert panel incorporates expertise from multiple domains 9
Supplementary Figures and Tables 12
References 18

Supporting materials and methods

Collection of expert opinions

We applied a WoC approach to collect information on the potential adverse effects of ENM exposures. We approached 79 established experts in the nanosafety community via email. The experts were provided with a general description of the project and asked about their interest in contributing to it. The experts showing interest towards the project were then provided with a table summarizing the 134 ENMs available in a previously published toxicogenomics data collection[1] together with their main physicochemical characteristics and the toxicological endpoints selected for this study. Details of the ENMs and endpoints are available in the file provided to the experts (Supplementary File 2) while an overview of the ENMs is provided in Figure S1.

The experts were asked to indicate a potential connection between each of the ENMs and the 18 endpoints in toxicologically relevant experimental setups. The original endpoints included "overall hazardous" which was eventually left out and replaced by the computed concern score "overall concern" which considers the values of the other inferred scores (see *Response modeling and inference of concern levels*). To be conservative against false negatives, the experts were asked to indicate the absence of a connection between an ENM and an endpoint only if they were convinced the connection does not exist. Otherwise, a possible connection was to be indicated. While the experts were encouraged to provide an answer even if they had no specific expertise regarding the exact ENM or endpoint, they could also provide an indefinite response (i.e., "not sure").

Field of study analysis

We used the Semantic Scholar Application Programming Interface (API)[2] (https://api.semanticscholar.org/api-docs) to retrieve the list of all publications authored by each expert. We extracted the keywords associated with the fields of study for each paper. We retrieved a total of 3601 papers and collected 23 unique field of study keywords. The median number of keywords per paper is 3. We built a binary matrix where each row represents a paper and each column a keyword. Each cell with coordinates (i, j) in the matrix has a value of 1 if the i-th paper was annotated with the j-th keyword. To prioritize the keywords that are likely to be significant for distinguishing and characterizing the papers, we assigned a relevance score to each keyword based on its inverse document frequency (IDF). In brief, IDF measures the importance of a keyword across the collection of papers. Common keywords among many papers receive a lower IDF score, while terms that are less frequent and appear associated with fewer papers receive a higher IDF score. The IDF of a keyword t in the corpus of papers D is computed as the logarithm of the total number of papers divided by the number of papers associated with t: $\mathrm{IDF}(t, D) = \log\left(\frac{|D|}{1+|D_t|}\right)$, where $|x|$ is the number of elements in set x and D_t is the set of papers that are associated with the keyword t. The addition of 1 in the denominator ensures numerical stability when D_t is empty.

To facilitate interpretability of the results we reduced the dimensionality of the IDF matrix using non-negative matrix factorization (NMF)[3]. Namely, let X be the 3601×23 binary matrix that contains the IDF scores for each paper and each keyword, we factorize $X \approx WH$ with both

W and H being non-negative. In more detail, H is a $k \times 23$ matrix and its rows contain a set of basis vectors used to approximate the matrix X, while the rows of the $3601 \times k$ rows of W store the weights to combine the basis vectors to approximately reconstruct the original matrix X. Here, we set $k = 6$.

To quantify the contribution of each keyword to each component, we normalized each row of H to unit norm and ranked the keywords from the highest contribution to the lowest and plotted the top 4 keywords in Figure 1A. Similarly, to build the scientific signature for each expert annotator, we normalized the rows of matrix W to unit norm and averaged the rows of W that correspond to the papers authored by each expert. Finally, we clustered the scientific profiles by hierarchical clustering with the Ward method and cut the corresponding dendrogram to 0.5 to obtain an assignment of experts into cohesive groups, represented in Figure 1B.

Response modeling and inference of concern levels

We aimed to assign a concern label to each ENM for each individual endpoint as well as an overall concern level per ENM across all the endpoints. However, the data collected from the experts was unbalanced in the number of responses for each ENM and for each

endpoint. To mitigate this, and to obtain more robust estimations, we employed a Bayesian hierarchical model.

We followed an approach similar to Whitehill *et al.* [4] and considered a latent variable that models the overall level of "concern" of each ENM, given that it is derived from the collective opinion of a panel of experts instead of experimental assays. The overall concern level summarizes how likely it is for an ENM to be considered harmful across all the surveyed endpoints. For each pair of ENM and endpoint considered, we assume a hidden binary label $Z_{ij} \sim \text{Bernoulli}(p_i)$ that represents whether ENM i is harmful for endpoint j, based on the overall concern level p_i. In addition, to model the survey responses, we assumed that not all experts share the same expertise and that not all endpoints are studied at the same depth (we call this the "difficulty" of an endpoint). Formally, the probability of expert k answering $y_{ijk} \in \{0,1\}$ for ENM i being harmful for endpoint j is:

$$
\begin{aligned}
p(y_{ijk}|p_i, \alpha_k, \beta_j) &= \sum_{Z_{ij}} p(y_{ijk}, Z_{ij}|p_i, \alpha_k, \beta_j) \\
&= \sum_{l=0}^{1} p(y_{ijk}|Z_{ij} = l, \alpha_k, \beta_j)p(Z_{ij} = l|p_i) \\
&= p(y_{ijk}|Z_{ij} = 0, \alpha_k, \beta_j)p(Z_{ij} = 0|p_i) \\
&\quad + p(y_{ijk}|Z_{ij} = 1, \alpha_i, \beta_j)p(Z_{ij} = 1|p_i) \\
&= \sigma_{\alpha_k\beta_j}^{(1-y_{ijk})}\left(1-\sigma_{\alpha_k\beta_j}\right)^{y_{ijk}}(1-p_i) + \sigma_{\alpha_k\beta_j}^{y_{ijk}}\left(1-\sigma_{\alpha_k\beta_j}\right)^{(1-y_{ijk})} p_i
\end{aligned}
$$

where $\sigma_{\alpha_k\beta_j} = \frac{1}{1+e^{-\alpha_k\beta_j}}$ is the probability that expert k correctly labels ENM i in endpoint j, given its hidden label Z_{ij}.

In more detail, the difficulty of an endpoint is modeled as a non-negative score $\beta_j \in [0, +\infty)$. For values of β_j close to 0, the likelihood of any expert to predict the correct concern label of an ENM for endpoint j approaches 0.5, indicating a more difficult endpoint to predict. The higher the value of β_j, the easier it is for experts to correctly predict the concern label of any ENM for endpoint j. The model also considers the level of expertise of each survey participant, using the parameter $\alpha_k \in (-\infty, +\infty)$. Higher values of α_k imply that expert k is more likely to predict the correct label of ENM concern for the various endpoints, while smaller values (towards 0) imply less accuracy in the predictions of the k-th expert. Conversely, negative values of α_k imply chances lower than 0.5 for expert k to correctly predict concern labels. This phenomenon can be interpreted either as wrong beliefs (a bias), or an adversarial (malicious) behavior of expert k. For parameters β_k and p_k, we used a non-informative prior distribution, while for α_k we used a weakly informative prior distribution assuming cooperative behavior from the experts. Specifically, $\alpha_k \sim \text{Normal}(1,1)$, $\beta_k = e^{\beta'_k}$ with $\beta'_k \sim \text{Normal}(1,1)$ and $p_k \sim \text{Beta}(1,1)$.

To infer the posterior distributions of the hidden labels Z_{ij} and variables p_i, α_k and β_j, we applied the framework of stochastic variational inference (SVI) which frames inference as an optimization problem[5]. We defined a parametric variational distribution for each latent variable in the model and applied the Adam[6] optimization algorithm to minimize the empirical lower bound (ELBO) of the log data likelihood[7]. We approximated the

posterior distribution of p_i with a Beta variational distribution $q_{\theta_i}(p_i)$, where the set of parameters θ_i correspond to the shape parameters of individual variables. Similarly, the posterior variational distributions $q_{\phi_k}(\alpha_k)$ and $q_{\phi_j}(\beta'_j)$, with $\beta_j = e^{\beta'_j}$ to ensure that β_j is non-negative, are Normal distributions, each parameterized by the corresponding mean and standard deviation. All these parameters were optimized by the Adam optimization algorithm to approximate the posterior distributions by minimizing the ELBO of the data likelihood. The model has been implemented using the probabilistic programming language (PPL) Pyro[8] using Python.

In addition, we infer the hidden labels Z_{ij} by sampling 1000 times the posterior $p(Z_{ij}|y_{ijk}, \alpha_k, \beta_j, p_i)$, automatically computed by Pyro, and choosing the most frequent value as the true hidden label for each pair of ENM i and endpoint j. Finally, each label assignment is associated with a corresponding uncertainty expressed as the frequency associated with the most frequent label, so that, e.g., a hidden label Z_{ij} that has been sampled 950 times with the value 1 and 50 times with value 0 is decided to be 1 with a certainty of 95%.

Comparison of the concern levels with experimental data

To assess the quality of our results, we compared the predicted concern scores with experimental data. We analyzed a harmonized dataset of 2896 samples of ENM cell viability assays collected from hundreds of peer-reviewed articles[9]. Briefly, the dataset includes several annotations about the ENMs (e.g., core material, coating, diameter, Zeta potential) and the experimental setup (e.g., cell type, concentration, exposure time, type of test performed, presence of positive controls). Despite containing multiple assays for cell viability, we focused on the most frequently reported MTT assay.

Since the concern levels are not related to any experimental setup, we considered all available combinations of cell type, ENM concentration and exposure time to build a distribution of the measured cell viability across all possible conditions. We identified matching ENMs between the experimental dataset and our predicted cytotoxicity concern scores, based on the core material and calculated the most frequent concern score for each type of ENM. Finally, we compared the distribution of cell viability measurements associated with each matched ENM with the corresponding concern level.

Toxicogenomic and descriptor data layers

Transcriptomic data and physicochemical properties used in this study were previously curated in Saarimäki et al.[1] with additional data from Gallud et al.[10]. These data are available in Zenodo[11] at https://doi.org/10.5281/zenodo.6425445 and in NCBI Gene Expression Omnibus (GEO) under accession number GSE148705, respectively. The advanced descriptors have been previously published in del Giudice et al.[12] and are available in the associated data & code repository[13] at https://doi.org/10.5281/zenodo.7674574.

The collection of toxicogenomics data contains 585 samples spanning 134 ENMs and multiple experimental setups. The ENMs from the original collection were aggregated based on the similarity of the supplier product codes, descriptions, and physicochemical characteristics and grouped by the core material. Since the experimental data collected contains various platforms, the number of measured genes differs across the experiments. We selected only human *in vitro* samples (294 samples) and the 7238 genes present in most platforms, allowing a max 10% missing values.

The physicochemical data layer included descriptors focusing on both molecular and electronic structure properties. A detailed description of the physicochemical descriptors and their computation was presented in del Giudice *et al.*[12]. Briefly, the computation of the ENM properties employed various models and methods. The liquid drop model was used to calculate attributes like the Wigner–Seitz radius, number of ENMs in agglomerates, the number of surface elements, the surface–volume ratio, and size-dependent interfacial thickness. Electronic structure descriptors were computed using density functional theory and semi-empirical quantum chemical methods, with Hamaker constants evaluated for bio-nano interactions via van der Waals forces. Atomistic descriptors were derived from chemical composition, potential energy, lattice energy, topology, size, and force vectors, providing insights into stability and interaction potentials. Additionally, properties such as band gaps, heat of formation, electronegativity, hardness, dispersion energy per atom, dipole moment, and static polarizability were calculated using advanced parameterization techniques and software.

To build the predictive classifier we considered those physicochemical properties that were not presented to the experts, thus the following measurements were removed: shape features (diameter, length, width, surface area, mean diameter in water and mean diameter in medium), zeta potential and purity. Although endotoxins presence/absence was one of the values included among the descriptors, this data was omitted given the endotoxin content is unique to specific experiments and ENM batches and is not always reported.

To avoid fragmenting the available data, we aggregated the human gene expression data coming from the different experimental conditions into a single transcriptional profile assigned to each ENM using either the median of the values for each gene or the absolute maximum fold-change. Eventually, 88 different ENMs were used to build the machine learning-based classifier, using those that are present in all three data layers (toxicogenomics, physicochemical and expert labels).

Machine learning classifiers

We set up several classification task instances depending on the data used (descriptors, gene expression or both), the type of aggregation for gene expression data (median or absolute maximum of fold-changes) and the class labels to predict (the ENM concern level for an endpoint or the overall concern). To establish a binary label also for the overall concern scores, we considered whether the approximate posterior distributions $q_{\theta_i}(p_i)$ have a mode either above (label 1) or below (label 0) 0.5. We estimated the uncertainty associated with the overall concern labels by first computing the differential entropy of each approximate posterior label distribution $h(p_i) = E\left[-log\left(q_{\theta_i}(p_i)\right)\right]$. We then

transformed the differential entropy of each overall concern label, h, with $1 - exp(h)$ to obtain an uncertainty value lying in the range [0,1], where larger values are associated with more certainty regarding the concern level.

For each classification task instance, we trained a gradient boosting classifier[14,15] with 5000 trees, each with a maximum depth of 50 levels, to allow the classifiers to learn the various interactions among the features. In addition, we weighed each sample with a score proportional to the uncertainty of the corresponding sample's concern label to allow the models to learn the most relevant features from the samples which were labeled with the most certainty.

For each classification task, we performed a 5-fold cross-validation repeated 10 times and measured the ROC-AUC on each test fold in turn to estimate the generalization capabilities of each model.

For each endpoint and the overall concern level, we defined two classification strategies. The first strategy was to train a classifier only on a single data view (either physicochemical properties or gene expression); while the second strategy integrated the two data views using either an early integration approach or a late integration approach[16].

The early integration strategy consisted of first concatenating the features of each data view and then training a single classifier on this composite feature space. This strategy enables the classifier to learn relevant feature interactions both within each single data view as well as between the two different data views. Due to the dimensionality of such integrated datasets, we trained an early integration classifier merging only the top 10 relevant features from the corresponding single view classification tasks. To avoid overfitting, we used the training folds of the cross-validation to train both the single view classifiers and the (top 10) early integrated classifier, each of those is then validated on the respective held-out fold.

The late integration strategy resembles ensemble learning, where each model is trained on a single data view and the predictions are aggregated to obtain a final answer across the views. Specifically, we trained a single view classifier on each data view and then built a new dataset, where the features correspond to the concatenation of all the predicted class-conditional scores from the single-view classifiers. Then, an integrative classifier is trained on this dataset, where the assumption is that the multi-view classifier would be able to catch and correct any systematic errors from the predictions of the single-view models. Also here, we exploited the structure of the cross-validation splits to reduce the chances of overfitting. In this case, the predicted label for each given ENM, e, is obtained by the model in which e was part of the held-out fold and was not used for training.

After each training repetition, we collected the top 10 most relevant features (genes). To measure feature relevance, we evaluated the overall information gain between the class label and each feature used to split the data across all the trees of the boosted classifier. The most recurring relevant features across all the repetitions of each classification task were reported.

Interactive viewer implementation

We implemented an interactive data explorer to empower users in exploring the current data interactively. The R Shiny[17] based implementation of the provided data explorer is focused on delivering a seamless, interactive and responsive user experience. Exploiting the capabilities of R packages as *InteractiveComplexHeatmap*[18] and *plotly*[19], the viewer is designed to handle dynamic interactions as users go through the exploration of the different plots representing the data. The data explorer and further instructions for its use are provided in GitHub alongside other code used in this study at https://github.com/fhaive/wisdom_of_the_crowds.

Supporting results

The expert panel incorporates expertise from multiple domains

In the absence of robust large-scale experimental data for many of the ENMs included in the collection, the reliability of the expert opinion can be improved by including diverse expertise and using data-driven approaches that support consensus-building without the influence of biased group dynamics. Hence, we compiled a panel of nanosafety experts with specific competence spanning various fields, ensuring richness of perspectives and breadth and depth of knowledge, and mitigating the risk of inherent biases that may arise from a homogenous group. With 17 expert opinions, our panel size aligns with the suggestion from Rowe and Wright (2001)[20] for structured expert elicitation. Moreover, Mannes et al. (2014)[21] showed that smaller, well-selected expert groups can outperform larger, less specialized ones in these types of tasks.

The diversity of the expert panel was further clarified by characterizing their individual expertise profile based on all the available publications associated with each expert. We consolidated all the fields of expertise into six components that summarize the possible contributions of each publication (Figure 1A). The components are expressed as the combination of the scientific fields relevant for each of them. Figure 1A reveals a clear mapping of each component to a specific discipline. Notably, component 5 highlights medicine (which includes toxicology in this case) as the main contributor with minor contributions from engineering and psychology, while component 3 displays a greater degree of heterogeneity, comprising engineering, physics, and computer science in more equal proportions. These fields are, however, closely related and largely overlapping, especially within the context of synthesis and characterization of chemicals and ENMs, where computation of numerical descriptors and binding affinity simulations are performed.

We then summarized the contribution fields represented by all the publications from each expert to obtain individual scientific profiles (Figure 1B). These profiles were obtained by summarizing the scientific context of each paper they authored. This analysis

underscores the multi-faceted nature of the field of ENMs safety assessment, reinforcing the need for a varied group of experts. A heterogeneous group not only enhances the accuracy of annotations but also enriches the collective intelligence, allowing a broader spectrum of expertise that better reflects the complexity of real-world scenarios than possessed by individuals. In harnessing the power of the wisdom of crowds, the incorporation of diverse knowledge and expertise is fundamental for ensuring a more robust and nuanced understanding of ENMs and their potential health effects.

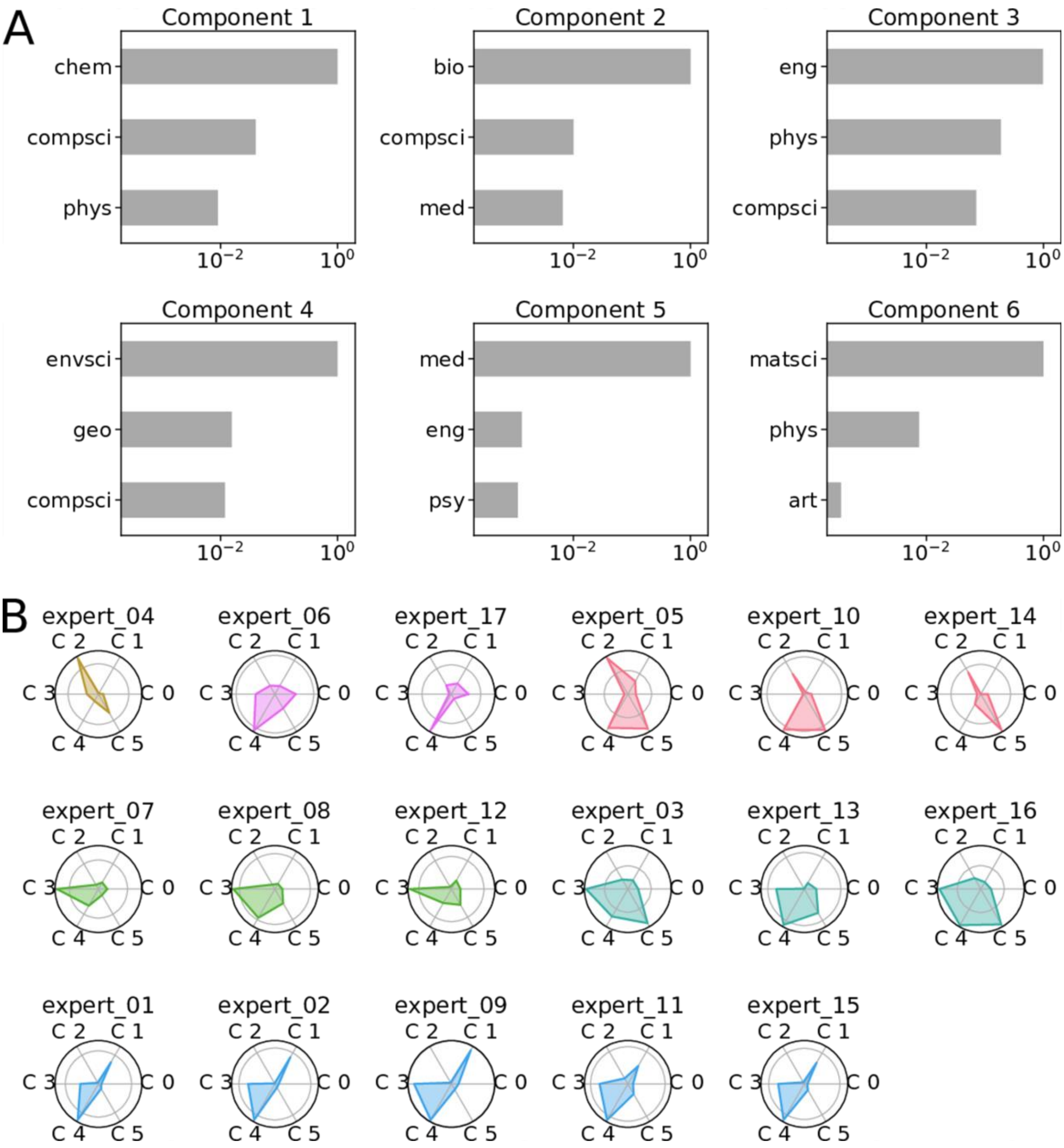

Figure 1. (A) Visual representation of how the collective literature published by the expert annotators can be decomposed into six different components (plotted in log_{10} scale to enhance visibility). Each component is linked to various scientific fields, highlighting the

diverse nature of expertise relevant to the publications. Abbreviations: chem = chemistry, compsci = computer science, phys = physics, bio = biology, med = medicine, eng = engineering, envsci = environmental science, geo = geology, psy = psychology, matsci = material science (B) The scientific profiles of each expert annotator. To facilitate comparisons, the profiles are clustered and assigned distinct colors. The shape of each plot emphasizes commonalities among experts in the same group and reveals differences among those in different clusters, highlighting patterns and variations in their expertise.

Supplementary Figures and Tables

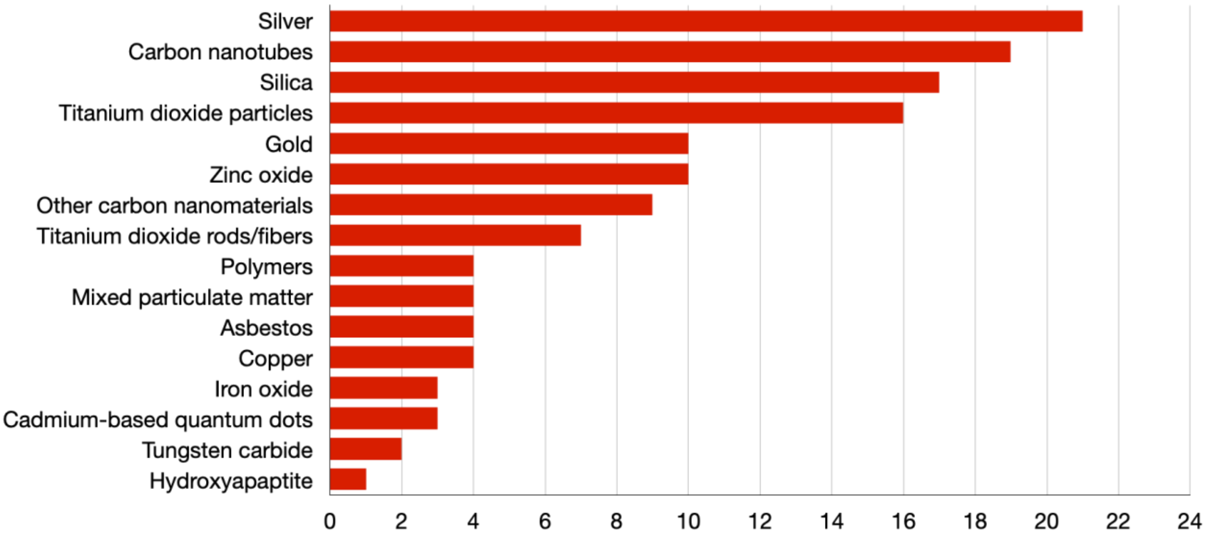

Figure S1. Number of distinct nanomaterials under each category defined by core material/type.

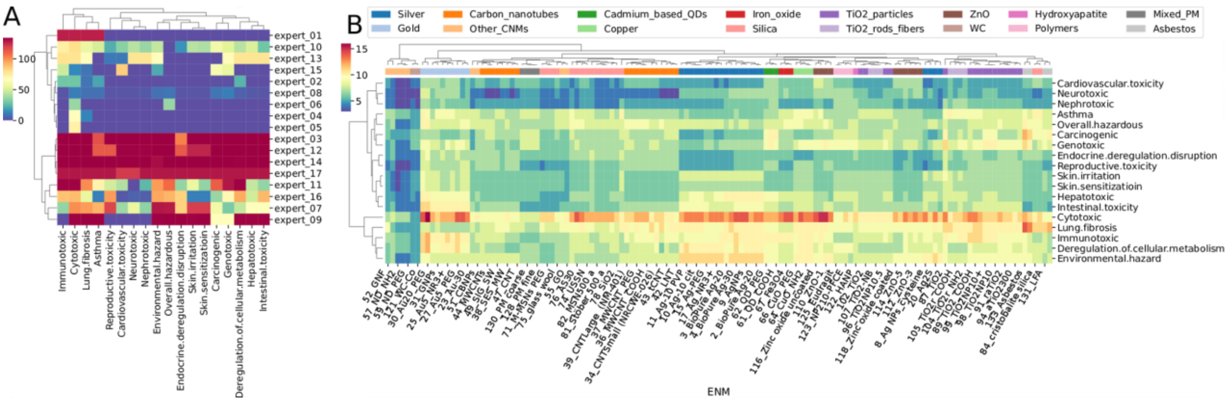

Figure S2. A) the number of definitive (positive/negative) responses from each expert for each endpoint. B) The number of total definitive responses by ENM and endpoint.

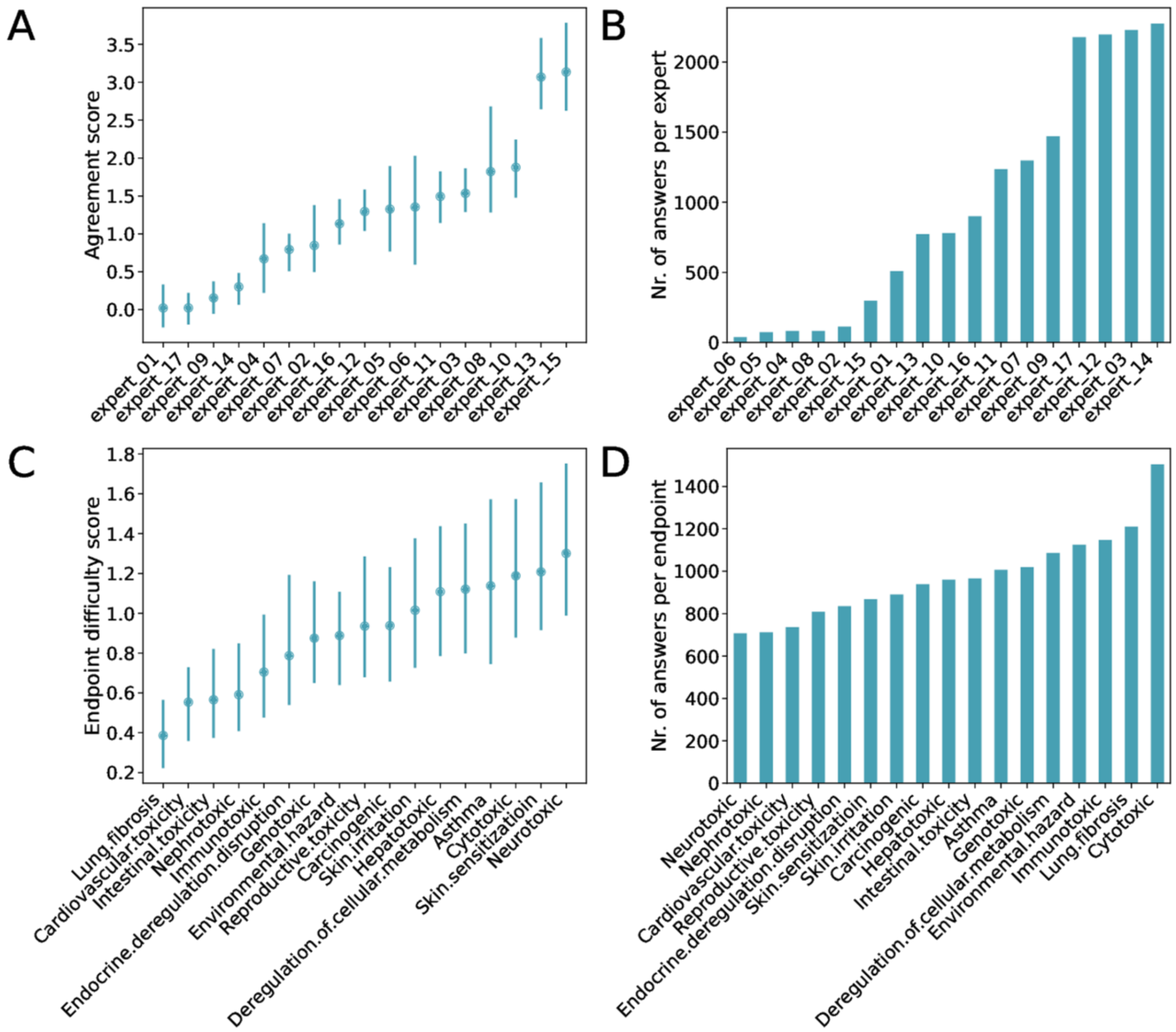

Figure S3. (A-B) 95% Highest Density Interval (HDI) of Posterior Distribution for Inferred Expert Agreement Scores of experts in labeling ENMs based on the estimated hidden labels (A). Higher scores indicate a greater likelihood of correct labeling. By comparison, the plot on the right summarizes the count of instances answered by each expert, indicating that agreement scores are not related to the quantity of answers provided by the experts (B). (C-D) 95 % Highest Density Interval (HDI) of Posterior Distribution for Inferred Endpoint Difficulty Scores of the posterior distribution (C), showing the inferred scores for endpoint difficulty. Lower values for endpoints indicate greater difficulty for experts in labeling, reflecting less consensus among the annotators. Also in this case, endpoint difficulty scores exhibit a weak association with the quantity of answers provided by the experts (D).

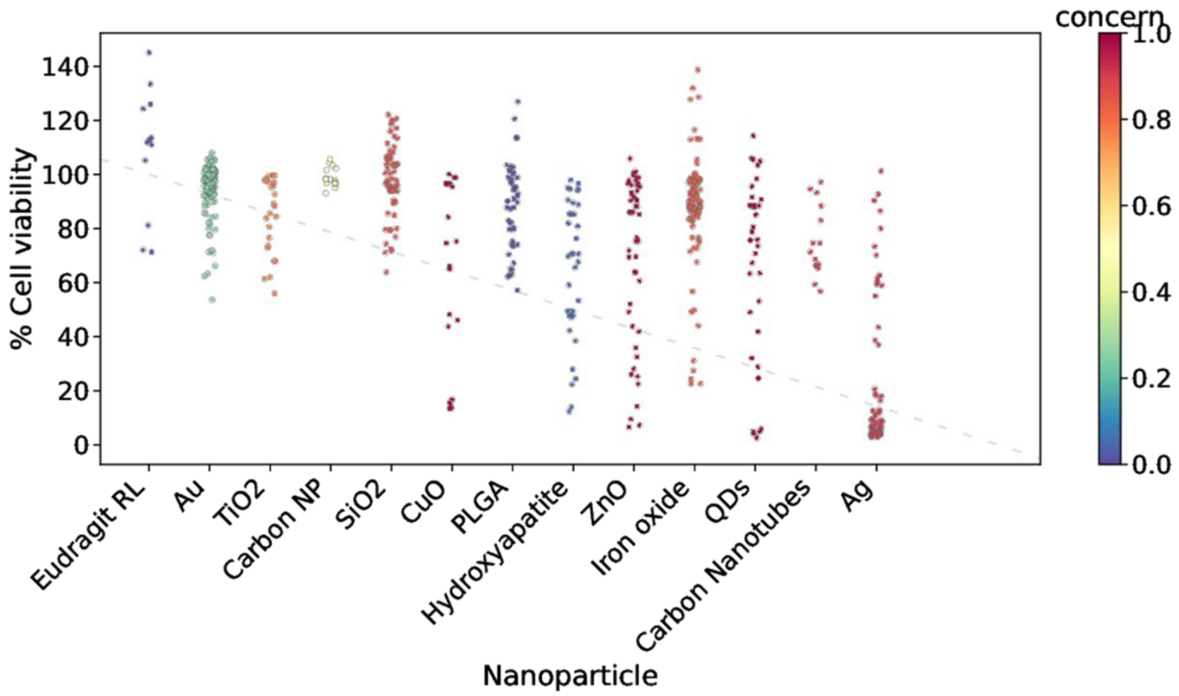

Figure S4. Comparison of predicted cytotoxicity concern levels and experimental evidence derived from cell viability assays. ENMs have been grouped by their core material/type and the distributions of cell viability across all viability assays performed on the distinct types of ENMs are shown. The grouped ENMs are sorted based on the mode (i.e., the most frequent value) of the distributions in ascending order, while the color represents the corresponding cytotoxicity concern score (color scale for concern with 0 indicating low concern, 1 high concern). This means that, on average, cell viability is consistent with the corresponding computed concern scores.

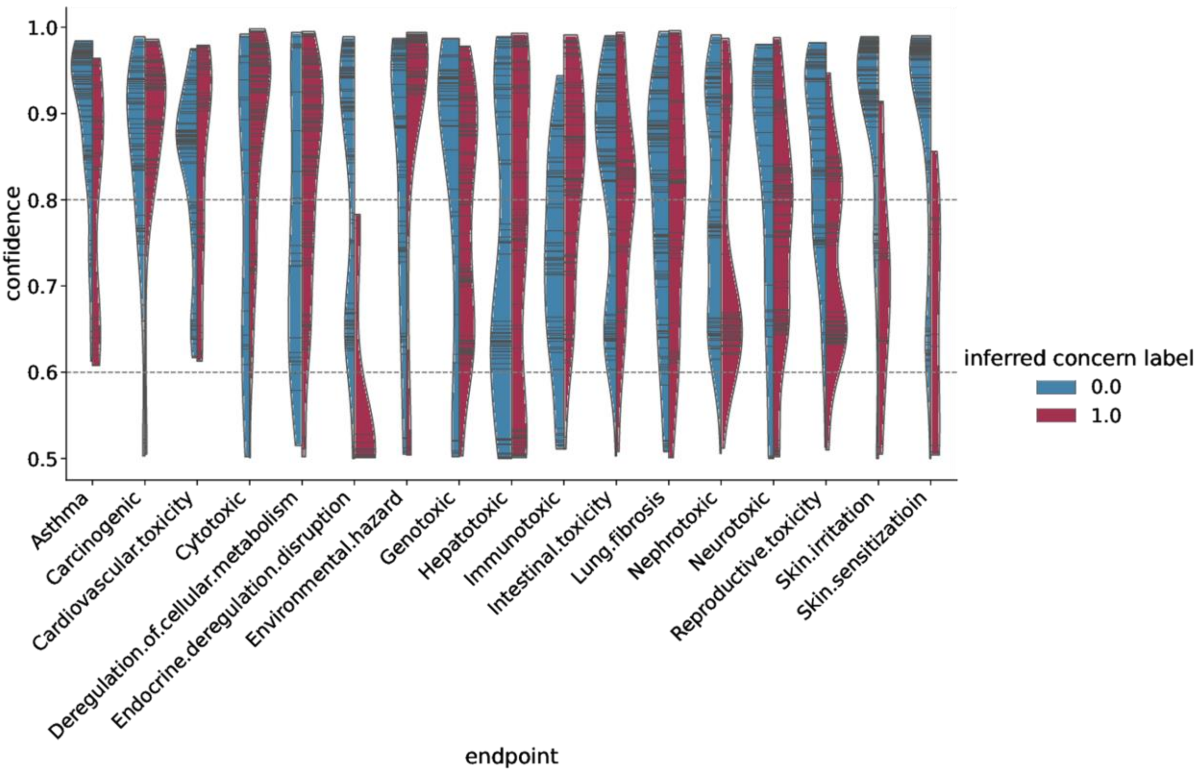

Figure S5. Violin plots illustrating the distribution of uncertainty in concern label assignment for each endpoint. Blue and red densities represent non-concerning (label 0) and concerning (label 1) ENMs, respectively. Individual ENMs are represented by lines within the density plots. The horizontal dashed lines divide the confidence levels into three groups: low confidence (< 0.6), mild confidence (0.6 - 0.8) and high confidence (> 0.8).

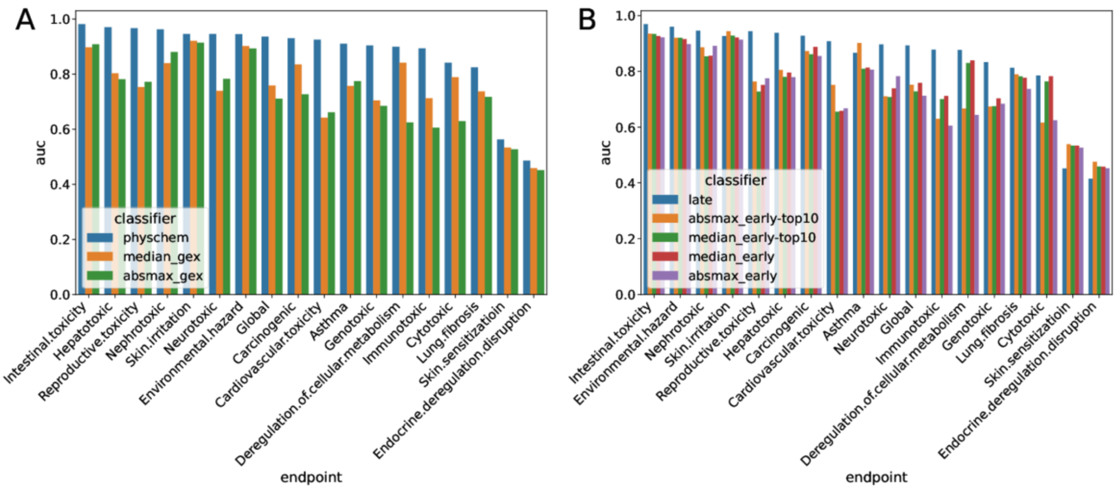

Figure S6. Classifier ROC-AUC. (A) Depiction of the single view classifier performance. The classifier based on ENM descriptors outperforms the one using transcriptomic features for all endpoints. (B) Multi-view classifier performance. The multi-view classifier based on the

late integration strategy has the highest performance for most of the endpoints over the early integration and single-view classification strategies.

Table S1: Top 10 most relevant genes for each endpoint.

**gene**	**endpoints**
GNB2	Cytotoxic, Deregulation of cellular metabolism, Genotoxic, Overall, Immunotoxic, Neurotoxic
MT1G	Environmental hazard, Hepatotoxic, Intestinal toxicity, Nephrotoxic, Reproductive toxicity, Skin irritation
DEXI	Genotoxic, Overall, Hepatotoxic, Immunotoxic, Neurotoxic
RAD52	Carcinogenic, Genotoxic, Overall, Immunotoxic
BIN3	Cardiovascular toxicity, Deregulation of cellular metabolism, Genotoxic, Immunotoxic
SLC30A1	Environmental hazard, Hepatotoxic, Intestinal toxicity, Nephrotoxic
MT1F	Environmental hazard, Intestinal toxicity, Nephrotoxic, Skin irritation
MT2A	Environmental hazard, Intestinal toxicity, Nephrotoxic, Skin irritation
RETREG2	Environmental hazard, Hepatotoxic, Intestinal toxicity, Nephrotoxic
RCC1L	Genotoxic, Overall, Immunotoxic, Neurotoxic
MFSD5	Cardiovascular toxicity, Nephrotoxic, Reproductive toxicity
SYNGR2	Cytotoxic, Deregulation of cellular metabolism, Immunotoxic
NAAA	Deregulation of cellular metabolism, Immunotoxic, Neurotoxic
THOC1	Environmental hazard, Intestinal toxicity, Nephrotoxic
PACSIN2	Asthma, Lung fibrosis
ESRP1	Asthma, Lung fibrosis
ZNF408	Cardiovascular toxicity, Genotoxic
HS1BP3	Environmental hazard, Nephrotoxic
AMDHD2	Environmental hazard, Intestinal toxicity
MAP3K5	Environmental hazard, Intestinal toxicity
PRPSAP1	Genotoxic, Reproductive toxicity
ZNF76	Overall, Neurotoxic
PEX7	Overall, Neurotoxic
B4GALT7	Overall, Hepatotoxic
AP5Z1	Hepatotoxic, Reproductive toxicity
NEO1	Lung fibrosis, Reproductive toxicity
BTN2A2	Nephrotoxic, Skin irritation
CAPN10	Neurotoxic, Reproductive toxicity
SPRY2	Asthma
CLK4	Asthma
PORCN	Asthma
NES	Asthma
ARNT2	Asthma
ZBED5	Asthma

GRAMD4	Asthma
COPS4	Asthma
TMEM185B	Carcinogenic
VRK3	Carcinogenic
CHPT1	Carcinogenic
ATG101	Carcinogenic
SLC16A2	Carcinogenic
ACTR1B	Carcinogenic
ALDH9A1	Carcinogenic
SLC1A5	Carcinogenic
PRMT1	Carcinogenic
INPP1	Cardiovascular toxicity
ECE1	Cardiovascular toxicity
TMEM53	Cardiovascular toxicity
DYRK3	Cardiovascular toxicity
KIAA0930	Cardiovascular toxicity
RGP1	Cardiovascular toxicity
PTPN14	Cardiovascular toxicity
YIF1A	Cytotoxic
TMED1	Cytotoxic
TOP3B	Cytotoxic
NAGA	Cytotoxic
TNNC1	Cytotoxic
MRM2	Cytotoxic
STX6	Cytotoxic
PQBP1	Cytotoxic
GFRA3	Deregulation of cellular metabolism
CCDC170	Deregulation of cellular metabolism
ARL15	Deregulation of cellular metabolism
ARMH3	Deregulation of cellular metabolism
C2orf49	Deregulation of cellular metabolism
NCDN	Deregulation of cellular metabolism
PHF7	Environmental hazard
TSFM	Genotoxic
CFAP74	Genotoxic
TTC12	Genotoxic
ZNF157	Overall
RARRES1	Overall
PDE6D	Overall
PBXIP1	Hepatotoxic
ALG6	Hepatotoxic
NRF1	Hepatotoxic
DHX38	Hepatotoxic

SLC39A8	Immunotoxic
ENSA	Immunotoxic
USP46	Immunotoxic
APH1B	Intestinal toxicity
HAMP	Intestinal toxicity
SPAG4	Lung fibrosis
ACSF2	Lung fibrosis
HTRA2	Lung fibrosis
ZNF189	Lung fibrosis
CD14	Lung fibrosis
KYNU	Lung fibrosis
ARHGEF2	Lung fibrosis
KCNE5	Nephrotoxic
MARCHF6	Neurotoxic
ZDHHC18	Neurotoxic
RHOG	Neurotoxic
TMED3	Reproductive toxicity
GRIK3	Reproductive toxicity
GRK5	Reproductive toxicity
IRF1	Reproductive toxicity
MLLT11	Skin irritation
H3C8	Skin irritation
FAM111A	Skin irritation
TXNRD1	Skin irritation
POLR3G	Skin irritation
IER5	Skin irritation

References

(1) Saarimäki, L. A.; Federico, A.; Lynch, I.; Papadiamantis, A. G.; Tsoumanis, A.; Melagraki, G.; Afantitis, A.; Serra, A.; Greco, D. Manually Curated Transcriptomics Data Collection for Toxicogenomic Assessment of Engineered Nanomaterials. *Sci. Data* **2021**, *8* (1), 49. https://doi.org/10.1038/s41597-021-00808-y.

(2) Kinney, R.; Anastasiades, C.; Authur, R.; Beltagy, I.; Bragg, J.; Buraczynski, A.; Cachola, I.; Candra, S.; Chandrasekhar, Y.; Cohan, A.; Crawford, M.; Downey, D.; Dunkelberger, J.; Etzioni, O.; Evans, R.; Feldman, S.; Gorney, J.; Graham, D.; Hu, F.; Huff, R.; King, D.; Kohlmeier, S.; Kuehl, B.; Langan, M.; Lin, D.; Liu, H.; Lo, K.; Lochner, J.; MacMillan, K.; Murray, T.; Newell, C.; Rao, S.; Rohatgi, S.; Sayre, P.; Shen, Z.; Singh, A.; Soldaini, L.; Subramanian, S.; Tanaka, A.; Wade, A. D.; Wagner, L.; Wang, L. L.; Wilhelm, C.; Wu, C.; Yang, J.; Zamarron, A.; Zuylen, M. V.; Weld, D. S. The Semantic Scholar Open Data Platform. arXiv January 24, 2023. https://doi.org/10.48550/arXiv.2301.10140.

(3) Lee, D.; Seung, H. S. Algorithms for Non-Negative Matrix Factorization. In *Advances in Neural Information Processing Systems*; MIT Press, 2000; Vol. 13.

(4) Whitehill, J.; Wu, T.; Bergsma, J.; Movellan, J.; Ruvolo, P. Whose Vote Should Count More: Optimal Integration of Labels from Labelers of Unknown Expertise. In *Advances in Neural Information Processing Systems*; Curran Associates, Inc., 2009; Vol. 22, pp 2035–2043.
(5) Blei, D. M.; Kucukelbir, A.; McAuliffe, J. D. Variational Inference: A Review for Statisticians. *J. Am. Stat. Assoc.* **2017**, *112* (518), 859–877. https://doi.org/10.1080/01621459.2017.1285773.
(6) Kingma, D. P.; Ba, J. Adam: A Method for Stochastic Optimization. arXiv January 30, 2017. https://doi.org/10.48550/arXiv.1412.6980.
(7) Ganguly, A.; Jain, S.; Watchareeruetai, U. Amortized Variational Inference: A Systematic Review. *J. Artif. Intell. Res.* **2023**, *78*, 167–215. https://doi.org/10.1613/jair.1.14258.
(8) Bingham, E.; Chen, J. P.; Jankowiak, M.; Obermeyer, F.; Pradhan, N.; Karaletsos, T.; Singh, R.; Szerlip, P.; Horsfall, P.; Goodman, N. D. Pyro: Deep Universal Probabilistic Programming. *J. Mach. Learn. Res.* **2019**, *20* (28), 1–6.
(9) Labouta, H. I.; Asgarian, N.; Rinker, K.; Cramb, D. T. Meta-Analysis of Nanoparticle Cytotoxicity via Data-Mining the Literature. *ACS Nano* **2019**, *13* (2), 1583–1594. https://doi.org/10.1021/acsnano.8b07562.
(10) Gallud, A.; Delaval, M.; Kinaret, P.; Marwah, V. S.; Fortino, V.; Ytterberg, J.; Zubarev, R.; Skoog, T.; Kere, J.; Correia, M.; Loeschner, K.; Al-Ahmady, Z.; Kostarelos, K.; Ruiz, J.; Astruc, D.; Monopoli, M.; Handy, R.; Moya, S.; Savolainen, K.; Alenius, H.; Greco, D.; Fadeel, B. Multiparametric Profiling of Engineered Nanomaterials: Unmasking the Surface Coating Effect. *Adv. Sci.* **2020**, *7* (22), 2002221. https://doi.org/10.1002/advs.202002221.
(11) Saarimaki, L. A.; Federico, A.; Lynch, I.; Papadiamantis, A. G.; Tsoumanis, A.; Melagraki, G.; Afantitis, A.; Serra, A.; Greco, D. Manually Curated Transcriptomics Data Collection for Toxicogenomic Assessment of Engineered Nanomaterials, 2020. https://doi.org/10.5281/zenodo.6425445.
(12) del Giudice, G.; Serra, A.; Saarimäki, L. A.; Kotsis, K.; Rouse, I.; Colibaba, S. A.; Jagiello, K.; Mikolajczyk, A.; Fratello, M.; Papadiamantis, A. G.; Sanabria, N.; Annala, M. E.; Morikka, J.; Kinaret, P. a. S.; Voyiatzis, E.; Melagraki, G.; Afantitis, A.; Tämm, K.; Puzyn, T.; Gulumian, M.; Lobaskin, V.; Lynch, I.; Federico, A.; Greco, D. An Ancestral Molecular Response to Nanomaterial Particulates. *Nat. Nanotechnol.* **2023**, *18* (8), 957–966. https://doi.org/10.1038/s41565-023-01393-4.
(13) Giudice, G. del; Greco, D. Data & Code Repository for the Article "An Ancestral Molecular Response to Nanomaterial Particulates," 2023. https://doi.org/10.5281/zenodo.7674574.
(14) Friedman, J. H. Greedy Function Approximation: A Gradient Boosting Machine. *Ann. Stat.* **2001**, *29* (5), 1189–1232.
(15) Chen, T.; Guestrin, C. XGBoost: A Scalable Tree Boosting System. In *Proceedings of the 22nd ACM SIGKDD International Conference on Knowledge Discovery and Data Mining*; KDD '16; Association for Computing Machinery: New York, NY, USA, 2016; pp 785–794. https://doi.org/10.1145/2939672.2939785.
(16) Pavlidis, P.; Weston, J.; Cai, J.; Grundy, W. N. Gene Functional Classification from Heterogeneous Data. In *Proceedings of the fifth annual international conference on Computational biology*; RECOMB '01; Association for Computing Machinery: New York, NY, USA, 2001; pp 249–255. https://doi.org/10.1145/369133.369228.
(17) Chang, W.; Cheng, J.; Allaire, J. J.; Sievert, C.; Schloerke, B.; Xie, Y.; Allen, J.; McPherson, J.; Dipert, A.; Borges, B.; Software, P.; PBC; library), jQuery F. (jQuery library and jQuery U.; inst/www/shared/jquery-AUTHORS.txt), jQuery contributors

(jQuery library; authors listed in; inst/www/shared/jqueryui/AUTHORS.txt), jQuery U. contributors (jQuery U. library; authors listed in; library), M. O. (Bootstrap; library), J. T. (Bootstrap; library), B. contributors (Bootstrap; Twitter; library), I. (Bootstrap; plugin), P. N. K. (Bootstrap accessibility; plugin), V. T. (Bootstrap accessibility; plugin), D. L. (Bootstrap accessibility; plugin), S. C. (Bootstrap accessibility; plugin), C. O. (Bootstrap accessibility; PayPal; plugin), I. (Bootstrap accessibility; library), S. P. (Bootstrap-datepicker; library), A. R. (Bootstrap-datepicker; library), B. R. (selectize js; library), S. B. (selectize-plugin-a11y; library), D. I. (ion rangeSlider; library), S. S. (Javascript strftime; library), S. L. (DataTables; library), J. F. (showdown js; library), J. G. (showdown js; library), I. S. (highlight js; R), R. C. T. (tar implementation from. Shiny: Web Application Framework for R, 2024. https://cran.r-project.org/web/packages/shiny/index.html (accessed 2025-02-13).
(18) Gu, Z.; Hübschmann, D. Make Interactive Complex Heatmaps in R. *Bioinformatics* **2022**, *38* (5), 1460–1462. https://doi.org/10.1093/bioinformatics/btab806.
(19) Shalabh. Interactive Web-Based Data Visualization with R, Plotly, and Shiny. *J. R. Stat. Soc. Ser. A Stat. Soc.* **2021**, *184* (3), 1150. https://doi.org/10.1111/rssa.12692.
(20) Rowe, G.; Wright, G. Expert Opinions in Forecasting: The Role of the Delphi Technique. In *Principles of Forecasting: A Handbook for Researchers and Practitioners*; Armstrong, J. S., Ed.; Springer US: Boston, MA, 2001; pp 125–144. https://doi.org/10.1007/978-0-306-47630-3_7.
(21) Mannes, A. E.; Soll, J. B.; Larrick, R. P. The Wisdom of Select Crowds. *J. Pers. Soc. Psychol.* **2014**, *107* (2), 276–299. https://doi.org/10.1037/a0036677.
